# Expression and molecular regulation of non-coding RNAs in HPV-positive head and neck squamous cell carcinoma

**DOI:** 10.3389/fonc.2023.1122982

**Published:** 2023-03-29

**Authors:** Dandan Guo, Mei Yang, Shiyun Li, Weiwei Zhu, Meixin Chen, Jiayu Pan, Dan Long, Zhaohui Liu, Chunlin Zhang

**Affiliations:** Department of Otorhinolaryngology, Head and Neck Surgery, Affiliated Hospital of Zunyi Medical University, Zunyi, Guizhou, China

**Keywords:** human papillomavirus, head and neck squamous cell carcinoma, non--coding RNAs, microRNAs, long non-coding RNAs, circular RNAs

## Abstract

Head and neck squamous cell carcinoma (HNSCC) is the sixth most prevalent malignancy worldwide. Accumulating evidence suggests that persistent HPV infection is closely related to a subset of HNSCC types, and the incidence of human papillomavirus (HPV)-positive HNSCC has been annually increasing in recent decades. Although the carcinogenesis of HPV-positive HNSCC has not been completely elucidated, it has been well confirmed that E6 and E7, the main viral oncoproteins are responsible for the maintenance of malignant transformation, promotion of cell proliferation, and increase in tumor invasion. Moreover, compared with HPV-negative HNSCC, HPV-positive HNSCC shows some special clinical-pathological features, which are possibly related to HPV infection and their specific regulatory mechanisms. Non-coding RNA (ncRNA) is a class of RNA lacking the protein-coding function and playing a critical regulatory role *via* multiple complex molecular mechanisms. NcRNA is an important regulatory pattern of epigenetic modification, which can exert significant effects on HPV-induced tumorigenesis and progression by deregulating downstream genes. However, the knowledge of ncRNAs is still limited, hence, a better understanding of ncRNAs could provide some insights for exploring the carcinogenesis mechanism and identifying valuable biomarkers in HPV-positive HNSCC. Therefore, in this review, we mainly focused on the expression profile of ncRNAs (including lncRNA, miRNA, and circRNA) and explored their regulatory role in HPV-positive HNSCC, aiming to clarify the regulatory mechanism of ncRNAs and identify valuable biomarkers for HPV-positive HNSCC.

## 1 Introduction

Head and neck squamous cell carcinoma (HNSCC) is a malignancy originating from several anatomic sites, including the oral cavity, pharynx, larynx, and nasopharynx (1). There are a relatively high incidence and mortality of HNSCC worldwide (2), with approximately 664,700 new cases and 406,800 deaths annually (3, 4). The infection of high-risk HPV is an independent carcinogenesis factor of HNSCC besides traditional carcinogenic factors such as tobacco smoking and alcohol. Previous studies have shown that the ratio of HNSCC cases with HPV infection was 40%-80% in the United States, and 20%-90% in Europe (5, 6).

Although the pathogenesis of HPV-positive HNSCC has not yet been fully elucidated, it has been well proven that E6 and E7 are the main oncoproteins of high-risk HPVs, which play a crucial role in tumorigenesis and progression of HPV-positive HNSCC (7). E6 and E7 can inactivate tumor suppressor protein p53 and retinoblastoma protein (pRb) involved in the cell cycle, genome stability, and epigenetic modifications (8), as well as affect the mutation and epigenetic changes of the host genome (9). Growing research has shown that epigenetic alterations also exert significant effects on the molecular regulation of HPV-induced tumorigenesis and progression (10, 11). The epigenetic regulation includes histone post-translational covalent modifications and effects of non-coding RNA (12).

In the generation of a malignant phenotype, cancer genetics and epigenetics are inextricably linked (11, 13). Epigenetics generally leads to changes in gene expression without changing DNA sequence, such as DNA methylation and post-transcriptional gene modifications by ncRNAs, and epigenetic changes can induce deregulation of oncogenes and/or tumor suppressor genes (12, 14). Numerous studies have indicated that ncRNAs are involved in regulating the biological behaviors of HNSCC, such as the proliferation, invasion, and therapeutic resistance (15–18).

**Figure 1:**
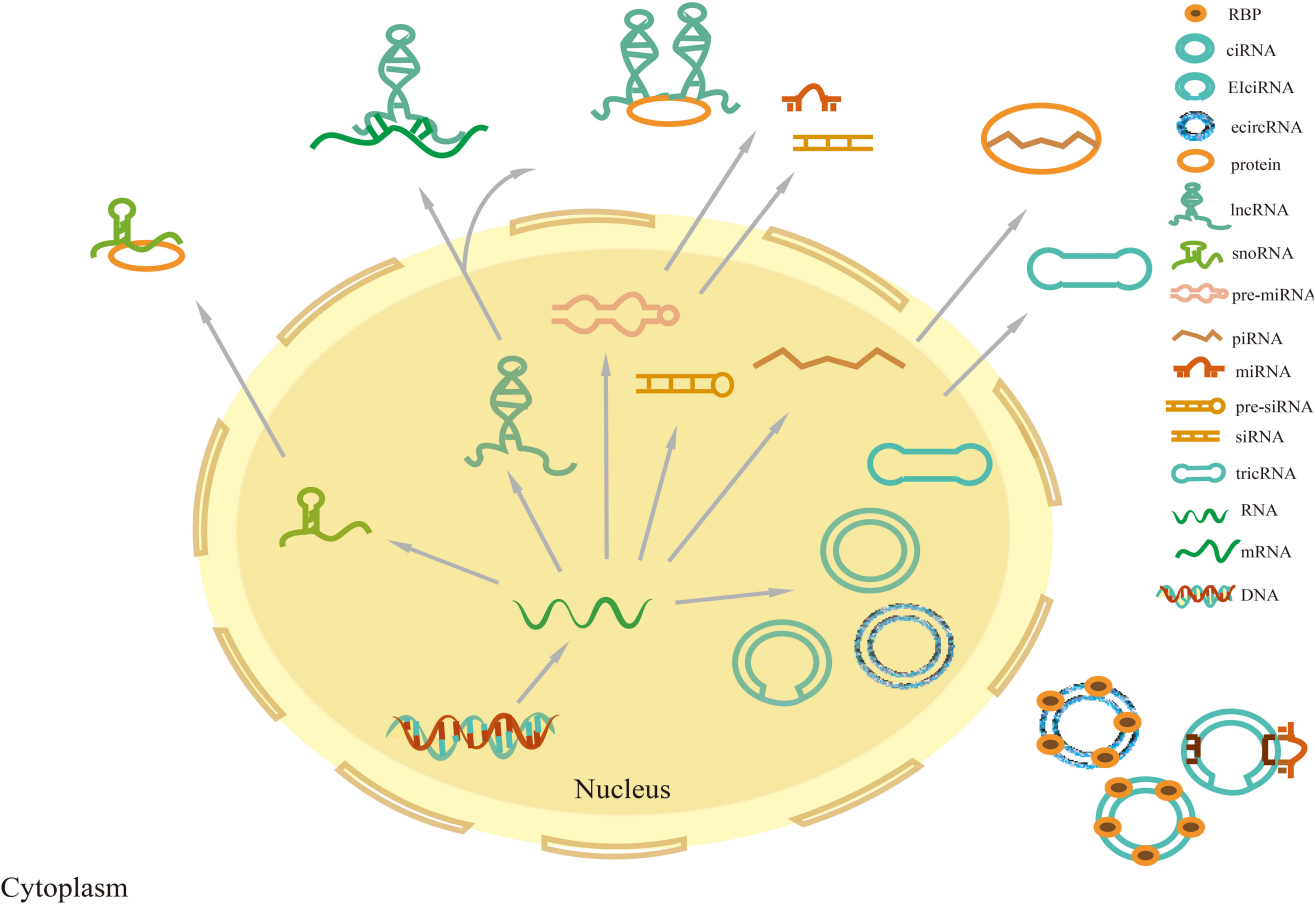
Classification of non-coding RNAs. Precursor miRNAs and siRNAs are transcribed and processed into mature miRNAs and siRNAs, respectively, which exert their function in the nucleus and cytoplasm. Precursor piRNAs are processed into mature piRNAs that form piRNA-PIWI complexes by combining them with PIWI proteins in the cytoplasm. TRNA intron splicing can lead to RNA cyclization, producing tricRNA. CircRNAs are divided into three main categories: intron-derived circular intronicRNAs (ciRNAs), exon-derived exonic circRNAs (ecircRNAs), and exon-intron circRNAs (EIcircRNAs). EIcircRNAs are mainly located in the nucleus, while ecircRNAs and tricRNAs are synthesized in the nucleus and will be exported to the cytoplasm. Most lncRNAs are located in the nucleus, and rarely encode proteins, while some are located in the cytoplasm.

NcRNAs are transcripts with nucleotide (nt) length less than 200, which are classified into long non-coding RNA (lncRNA), circular RNA (circRNA), microRNA (miRNA), P-element-induced wimpy testis interacting RNA (PIWI-Interacting RNA, piRNA), small nucleolar RNA (snoRNA), small interfering RNA (siRNA), ribosomal RNA, and tRNA depending on the nucleotide length (18) (**Figure 1**).

Available literature shows that ncRNAs are an important player in carcinogenesis (19, 20). MiRNAs generally bind to the 3′-untranslated region (UTR) of mRNA transcripts to participate in some biological processes (19). Some miRNAs can act as oncogenic factors or tumor suppressors in HPV-positive HNSCC (20–22). The function of miRNAs is biased by competing endogenous mutations of miRNA-binding sites (23). Besides, miRNAs are well-known downstream targets of lncRNAs. LncRNAs are primarily characterized by a 7-methylguanosine cap at the 5′-end, and a polyadenylated tail at the 3′-end (18). Although they do not encode proteins, lncRNAs can regulate gene transcription *via* multiple mechanisms, such as the competitive endogenous RNA (ceRNA) mechanism (24). CircRNAs are a new category of closed-loop ncRNAs and harbor several functional roles in the development of HNSCC *via* diverse molecular mechanisms, which mainly act as sponges that efficiently subtract miRNAs or proteins involved in oncogenesis (25). Furthermore, viral oncoproteins E6 and E7 have been reported to deregulate some ncRNAs, thereby modulating tumorigenesis and cancer progression. Thus, ncRNAs are valuable potential prognostic biomarkers for HPV-positive HNSCC (26).

HPV-positive HNSCC shows some special clinical characteristics, such as rapid proliferation, strong invasion, early lymph node metastasis, higher sensitivity to chemo- and radio-therapy, thus, a relatively favorable prognosis (15, 23, 27–30), which is related to the molecular regulation of ncRNAs (14, 31, 32). This review mainly focused on the expression of ncRNAs and addressed ncRNAs regulatory mechanisms in HPV-positive HNSCC, excluding siRNA, rRNA, and tRNA, as their intracellular function has already been validated. In addition, ncRNAs are promising biomarkers for inchoate discovery and prognostic prediction, and potential therapeutic targets in HNSCC.

## 2 MicroRNA

MiRNAs are endogenous ncRNAs of approximately 19-25nt in length, that are processed into precursor miRNAs in the nucleus and then transferred to the cytoplasm (19). MiRNAs can modulate the expression of cellular proteins, which function by binding to the 5′-UTRs and 3′-UTRs of their target mRNAs (19, 33). MiRNAs have been implicated predominantly in different stages of cell malignant transformation at transcriptional and translational levels (34). Specifically, the expression level of certain miRNAs has been associated with HPV infection. As HPV integrates into the host genome, the viral oncoproteins can modulate the expression of host genes and may also perturb the level of miRNAs (23, 35). Some miRNAs can induce abnormal cell cycle, affect cell apoptosis, or even alter genomic stability, thereby affecting the radiotherapy sensitivity (36). Moreover, miRNA expression level has been correlated with tumor stage, lymph node metastasis, radiotherapy resistance, and clinical prognosis of HNSCC (23, 37). Thus, miRNAs are considered prognostic and diagnostic markers in HPV-positive HNSCC.

### 2.1 Expression and significance of miRNAs in HPV-positive HNSCC

By now, a few studies have explored specific miRNA expression profiles in HPV-positive HNSCC tissues and cells (37–44), we summarize the miRNAs expression profile in premalignant and HNSCC tissues with HPV infection in **Supplemental Table 1**. The expression level and modulation status of these miRNAs are different between HPV-positive and HPV-negative samples. Besides, the distinctively expressed miRNA in HPV-positive HNSCC is significantly distinct from its counterpart in terms of assuming roles in clinical characteristics modulation. Furthermore, there are some differences in molecular mechanisms of miRNAs function between HPV-positive HNSCC and HPV-negative cases.

MiRNAs are distinctly expressed in HPV-positive HNSCC, which has been identified by various detection methods (40, 42, 45, 46). Lajer (42, 46) has first reported different miRNAs expressions in 51 patients with oral squamous cell carcinoma (OSCC) and pharyngeal squamous cell carcinoma (PSCC) by using microarray analysis. The researchers have then further revealed that the infection of HPV influenced 21 miRNAs, which might induce distinct clinical characteristics. Thereafter, many researchers have focused on miRNAs expression profiles in HPV-positive HNSCC, Gougousis et al. (45) have reported that miR-15, miR-16, miR-143, miR-145, and the miR106-363 cluster were overexpressed in HPV-positive oropharyngeal squamous cell carcinoma (OPSCC), Vojtechova and co-workers (40) have analyzed the differential expression by TaqMan real-time quantitative PCR (RT-PCR) array in HPV-positive and HPV-negative tonsillar tumors, in which 30 miRNAs were expressed in HPV-positive samples and 38 miRNAs were expressed in HPV-negative samples (**Supplemental Table 1**).

Next-generation sequencing (NGS) technology has been used for performing discrepant sequence alignment of genomes, which was developed based on RT-PCR and gene chips technology (47). The Cancer Genome Atlas (TCGA) data covers miRNA profiles and clinical details of HNSCC. MiRNA profiles obtained from TCGA data through NGS could screen differentially expressed miRNAs in HPV-positive and HPV-negative HNSCC tissues. Nunvar et al. (35) have reported that 70 and 116 specific miRNAs were differently expressed in HPV-positive and HPV-negative HNSCC, as identified by NGS.

The main significance of differently expressed miRNAs is that they are considered valuable biomarkers in HNSCC (48). Generally, miRNAs may represent novel biomarkers in HPV-positive HNSCC. House et al. (49) and Weiss et al. (50) have reported that miR-205-5p, miR-182-5p, and miR-133a-3p were overexpressed in HPV-positive OPSCC, and could be adapted as prognostic markers (49–51). Bersani et al. and Gougousis et al. (45, 51) have reported that miRNAs were correlated with distant tumor metastasis, invasion, and migration, even could discriminate tumor stages (2-4 of T stages) in HPV-positive Tongue squamous cell carcinoma (TSCC) and OPSCC. Moreover, miR-106a, miR-27a, and miR-9 have been intimately correlated with radiotherapy sensitivity in HPV-positive HNSCC, while miR-139-3p has been related to chemotherapy sensitivity in HPV-positive HNSCC (23, 52–54) (**Supplemental Table 2**).

### 2.2 Molecular regulation roles of miRNAs in HPV-positive HNSCC

As mentioned above, miRNAs have been associated with biological behaviors, thus, many researchers have begun to focus on the regulatory roles of miRNAs in HPV-positive HNSCC (15, 20). For example, Casarotto et al. (15) have identified that miR-375 and miR-139 could emerge as key players in modulating the occurrence of HPV-positive HNSCC. Luo et al. (55) have reported that miR-518a-5p and miR-605-5p could act as essential regulators in cell proliferation, apoptosis, tumor growth, and metastasis in HPV-positive HNSCC. Specifically, miRNAs act as essential regulators in biological processes by regulating target genes and key pathways involved in cell proliferation, apoptosis, tumor growth, epithelial-to-mesenchymal transition (EMT), and metastasis processes.

Moreover, miRNAs may perform a vital role in oncogenesis by acting as oncomiRNAs or tumor suppressor factors (18, 38, 43, 45, 52). Up to the present, miR-22, miR-27, miR-92a, miR-195, and miR-211 have been identified as oncogenic promotion miRNAs in HPV-positive HNSCC (18, 52, 56, 57), being involved in the occurrence of HNSCC through regulation of their target genes (22, 46, 54, 56, 58). For example, miR-21 and miR-155 can promote the proliferation and invasion of OPSCC cells *via* suppressing downstream target genes, such as phosphatase tensin homolog (PTEN) and signal transducer and activator of transcription (STAT) (54, 59, 60). On the other side, many miRNAs, including miR-16 and miR-17 (42, 43), have been identified as tumor suppressors in HPV-positive HNSCC (**Supplemental Table 3**). These miRNAs can revive the major tumor suppressor proteins p53, p21, and p16 (61), and transcription factors, such as E2F, and downregulate other multiple oncogenes, resulting in tumor suppression (62).

Recently, it has been proven that cell autophagy and immune response could influence the prognosis of HNSCC (63–65). Aranda et al. (66). have reported that miRNAs could regulate cell autophagy and immune response, thus, affecting the prognosis of HPV-positive HNSCC. Luo et al. (55) have identified that miR-380-5p, miR-338-5p, miR-16-1-3p, and miR-378a-3p could modulate favorable prognosis by activating an immune response in HPV-positive HNSCC. To better understand the regulatory role of miRNAs in HPV-positive HNSCC, the more in-depth research is in need.

Radiotherapy is one of the effective treatment strategies for patients with HNSCC. Nevertheless, increasing evidence shows that miRNAs are involved in the regulation of radiation response *via* their target genes (33, 67–71). According to the research of Fu et al. (72), genomic signatures of DNA repair can influence HPV-positive tumor radiation sensitivity, and miRNAs are probably involved in the regulation of DNA damages by modulating downstream genes. Zhang et al. (53, 69) have identified that miR-106a and miR-27a enhanced radiotherapy sensitivity in HPV-positive HNSCC by targeting *RUNX3* and *SMG1* levels, respectively. On the contrary, the overexpression of miR-125b can weaken the radiation sensitivity in HPV-negative cells *via* the reduction of ICAM2 (a molecule related to enhanced radio-sensitivity) (70).

### 2.3 The correlation between viral oncoproteins E6/E7 and miRNAs in HPV-positive HNSCC

Some miRNAs are distinctively expressed in HPV-positive HNSCC, which is regulated by HPV oncoproteins (18, 69, 73). The expression of miR-9, miR-20, miR-27a, miR-34a, miR-106a, and miR-363 have been related to E6/E7 in HNSCC tissues, and the expression change by these miRNAs is possibly induced by the modulation of E6 and E7 (18, 22, 29, 33, 53) (**Supplemental Table 3**).

**Figure 2:**
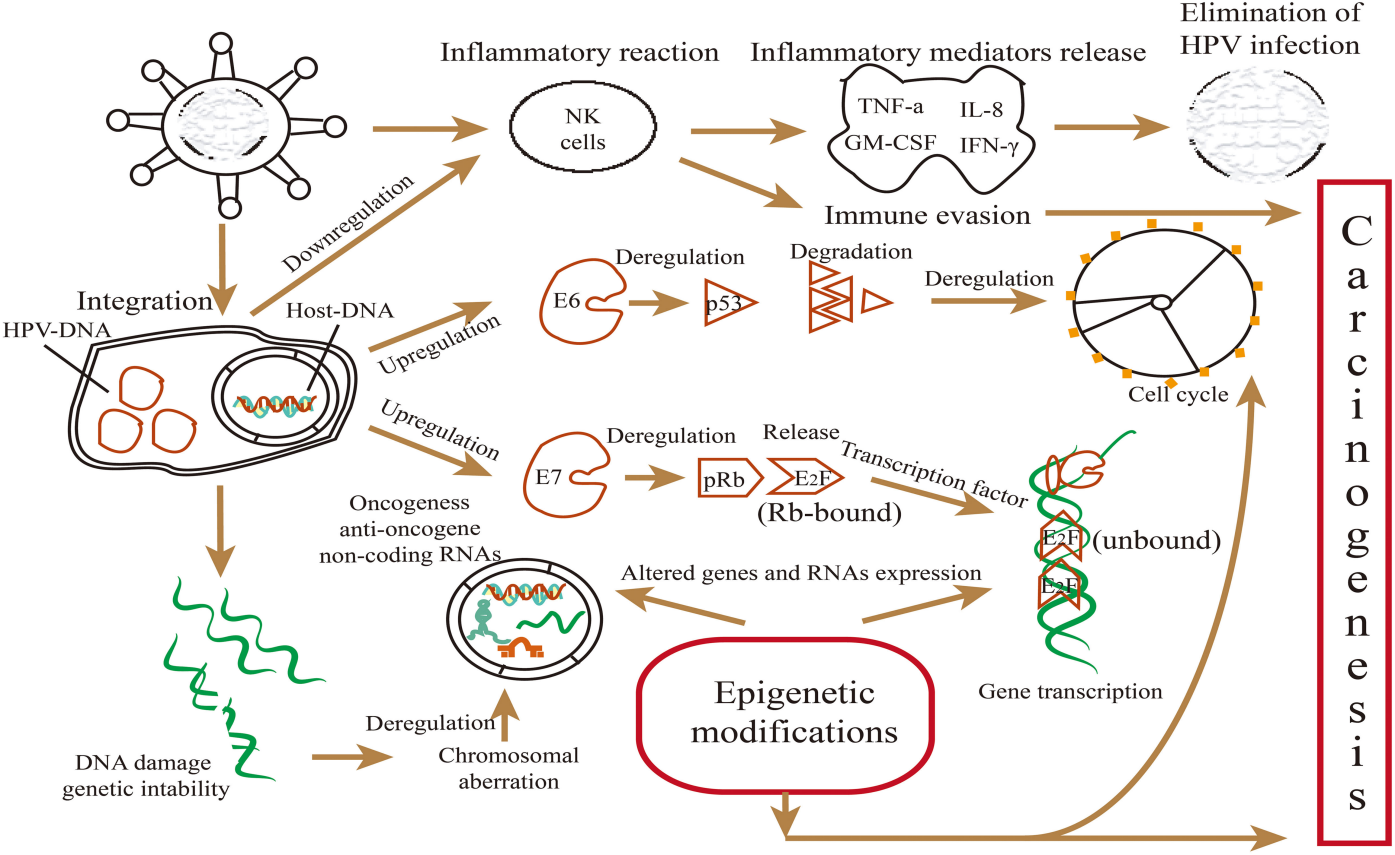
Main mechanisms involved in the malignant transformation and biological behaviors induced by viral oncoproteins E6/E7 in HPV-positive HNSCC. Oncoproteins E6 and E7 can inactivate tumor suppressor proteins p53 and pRb involved in the cell cycle, genome stability, and epigenetic modifications, as well as affect mutation and epigenetic changes of the host genome. E6 and E7 regulated the levels of miRNAs mainly by releasing p53 and E2F, thus, affecting miRNA expression, while miRNAs can activate or suppress downstream genes and key signal pathways involved in biological behaviors.

**Figure 3:**
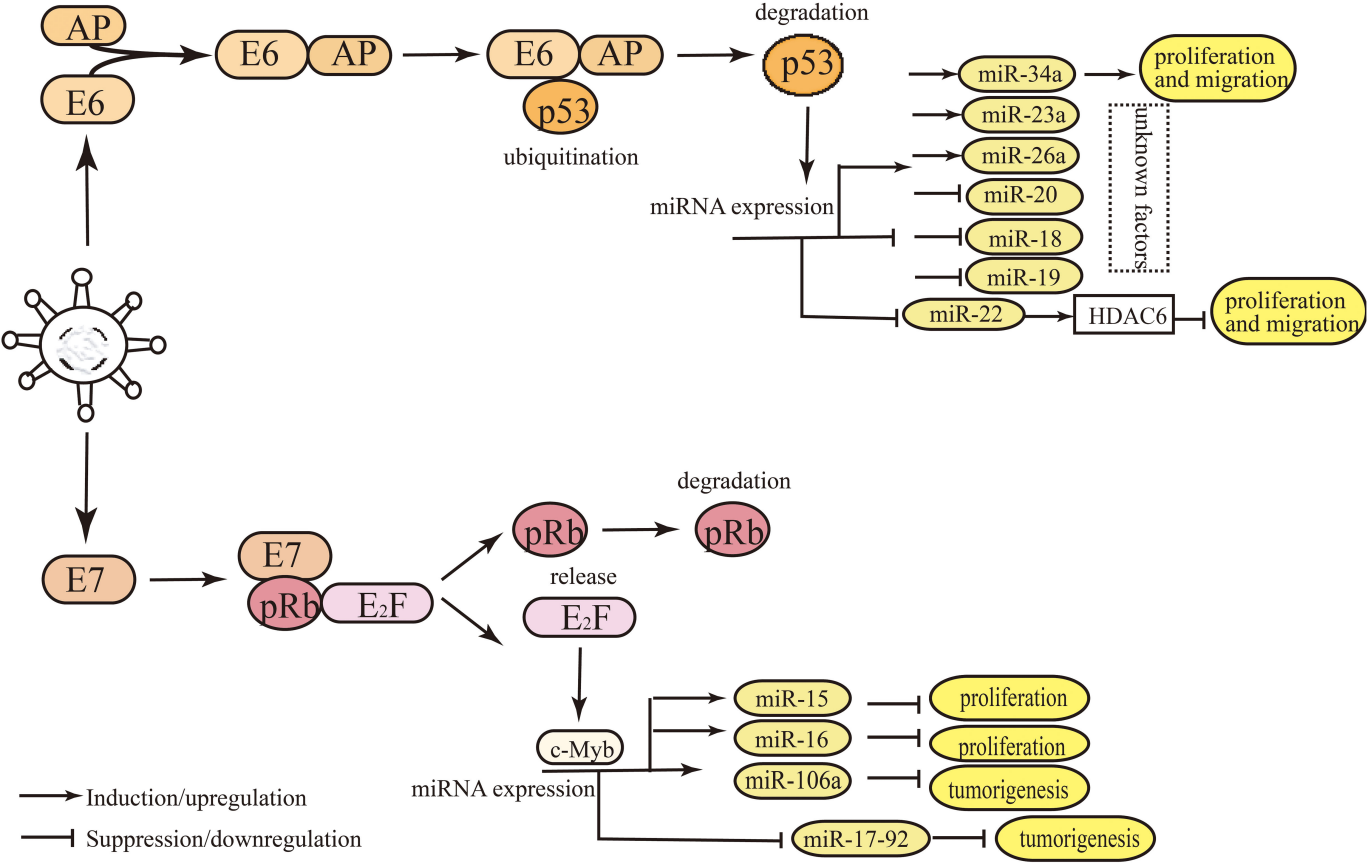
Main mechanisms of miRNAs expression regulation by E6/E7 in HPV-positive HNSCC and cervical cancer. E6 and E7 regulate the levels of miRNAs mainly by binding or releasing the transcription factors of miRNAs, such as c-Myc, p53, and E2F. The degradation of cellular transcription factor p53 is induced by E6, which can bind the promoter region of miRNAs. HPV E6 oncoprotein destabilizes p53, which contributes to the deregulated miRNAs. The degradation of pRb induced by E7 leads to the release of E2F from the pRB-E2F complex, and E2F binds to its binding site in miRNA promoter region, thus, affecting miRNA expression.

Research has illustrated the regulatory mechanism of viral oncoproteins in regulating miRNAs in HPV-positive HNSCC (16, 69, 74, 75). E6 and E7 regulate the expression level of miRNAs mainly by binding or releasing the transcription factors of miRNAs, such as c-Myc, p53, and E2F (76) (**Figure 2**). MiRNAs can activate or suppress downstream genes and key signal pathways involved in regulating biological behaviors (61, 77). However, most miRNAs regulated by E6 or E7 have been demonstrated in cervical cancer (**Figure 3**), and rarely in HNSCC. For instance, miR-363 is upregulated by E6, which deceases the expression level of *MYO1B*, thereby suppressing tumor progression in HPV-positive HNSCC cells (29, 74). E7 targets integrin β8 by upregulating miR-20a, thereby promoting migration and invasion of HPV-positive OSCC cells (75). Zhang et al. (53, 69) have revealed that viral oncoproteins affected radiation sensitivity by regulating miR-106a and miR-27a-3p levels and their downstream target genes *RUNX3* and *SMG-1* in HPV-positive HNSCC cells. According to Božinović et al. (28) and Nowek et al. (78), miR-9 level appeared to be upregulated by E6, and probably affects prognosis by promoting cancer‐associated fibroblast infiltration in HPV-positive OSCC. Nevertheless, the regulatory mechanism has not been elucidated completely, hence, further studies are necessary to explore the relationship between viral oncoproteins and miRNA signatures in HPV-positive HNSCC.

## 3 LncRNA

LncRNAs constitute a heterogenous group of RNA molecules exceeding 200nt in length without protein-coding function, which have been implicated in multiple biological processes by interacting with downstream RNAs, proteins, miRNAs, or circRNAs and even pseudogenes at transcriptional, and translation levels (24, 79).

**Figure 4:**
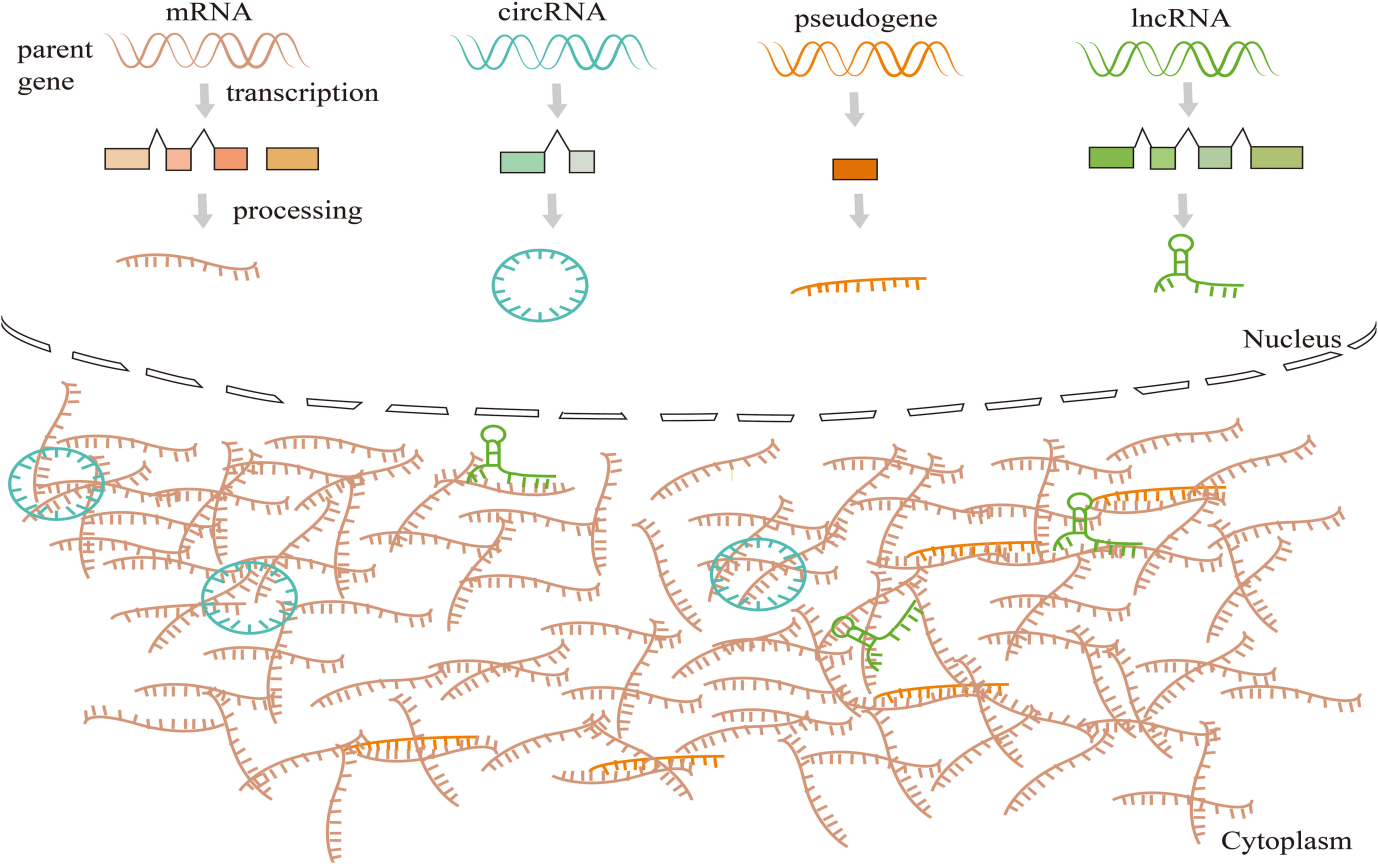
Competing endogenous RNAs (CeRNAs) networks and mechanisms. CeRNAs link the function of protein-coding mRNAs with miRNAs, lncRNAs, circRNAs, and pseudogenic RNAs. CeRNAs target binding (sponge) MREs (pseudogene transcripts, lncRNA, miRNAs, and circRNA) to revive the inhibition of downstream target genes, and this regulatory mode constitutes the ceRNA mechanism.

Many reports have indicated that lncRNAs could participate in cell proliferation, migration, and invasion, playing a key role in the tumor progression of HNSCC (15, 80). Moreover, lncRNAs can act as ceRNA, which sponges various RNAs to alter the expression level of target genes (81) (**Figure 4**), and then impacts various tumor behaviors (81, 82). HPV infection can lead to lncRNA aberrant expressions in host cells (83, 84), and deregulation of downstream molecules of some key signal pathways. Also, lncRNAs can serve as valuable biomarkers of HPV-positive HNSCC.

### 3.1 Expression and significance of lncRNAs in HPV-positive HNSCC

Emerging evidence demonstrates that discrepant lncRNAs are identified in clinical samples by RNA-sequencing, RT-PCR, and bioinformatics analysis tools (e.g., Cluster profiler package in R) (85–88). Thus, the aberrant expression of lncRNAs in HPV-positive HNSCC is summarized in **Supplemental Table 4**. For example, Wang et al. (86) have identified 131 distinctively expressed lncRNAs in the TCGA HPV-negative HNSCC dataset. Nohata et al. (85) have revealed 140 lncRNA transcripts alignment data generated from TCGA between HPV-positive and HPV-negative HNSCC *via* RNA-sequencing. Yang et al. (87) have reported 102 lncRNAs which were specifically expressed in HPV-positive HNSCC by using RNAs tools package in R. Furthermore, HPV infection has been associated with lncRNA expression (83, 85, 87, 89), Kopczyńska et al. (83) and Haque et al. (88) have demonstrated an array of differentially expressed lncRNAs in HPV-positive vs. HPV-negative HNSCC (**Supplemental Table 4**).

LncRNAs have been closely associated with some clinical characteristics. Kolenda et al. (90) have confirmed that the expression level of EGOT was related to age, N-stage, and the malignant phenotype in HPV-positive pharyngeal cancer. Guo et al. (91) and Kopczyńska et al. (83) have reported that PRINS and TTTY15 were upregulated and positively associated with a favorable prognosis of HPV-positive HNSCC (**Supplemental Table 5**). Moreover, aberrant lncRNA expression has been related to chemoradiotherapy resistance in HNSCC (92–94). As illustrated by Song et al. (92), lnc-IL17RA-11 exhibited a strong correlation with radiotherapy efficacy in HPV-positive HNSCC. Additionally, Fang et al. (93) and Zhang et al. (94) have demonstrated that lncUCA1 and lncWISP1 were related to radiation resistance in HPV-negative HNSCC (**Supplemental Table 5**).

### 3.2 Molecular regulation roles of lncRNAs in HPV-positive HNSCC

Some lncRNAs have been proven to play a role in the development and progression of HPV-positive HNSCC (18, 82). For instance, Ma et al. (82) and Dias et al. (95) have revealed that lincRNA-p21, HOTAIR, PROM1, and CCAT1 probably played a critical role in the development, invasion, and metastasis of HPV-related tumors. Further study has shown that lncRNAs could interact with miRNAs and regulate mRNAs expression (16, 80, 96). For example, lncRNA BLACAT1 promoted proliferation and invasion by sponging miR-142-5p in OSCC (80). However, there is little information about the tumor-suppressive effects of lncRNAs in HPV-positive HNSCC. Sannigrahi et al. (18) have reported that MEG3 performed a role of tumor suppression, probably through the promotion of cellular apoptosis by upregulating their target genes *GRP78* and *IRE1* (97).

Aberrant expression of lncRNAs also influences the survival prognosis of HPV-positive HNSCC (85, 91, 98, 99). Research has indicated that clinical prognosis is affected by autophagy and tumor immune response (99, 100), and lncRNAs influence prognosis by modulating the genes or signaling pathways involved in autophagy and immune activities. According to Guo et al. (99), TTTY15 affected the prognosis probably by upregulating autophagy-related protein (BECN1 and LC3) or activated autophagy-related pathway (STAT3-BRCA1 pathway) in HPV-positive HNSCC. Another study has reported that lncRNAs expression could regulate tumor immune infiltration, and a lower expression level of lncIRLPS might trigger a stronger immune response, leding to a better prognosis (98). However, additional clinical data are required to confirm the prognostic value of these lncRNAs in HPV-positive HNSCC.

Comparable to miRNAs, lncRNAs probably function as molecular sponges to attenuate downstream genes involved in gene stability in HPV-positive HNSCC (92, 94, 101). Thus, lncRNAs can significantly enhance radiation sensitivity, lnc-IL17RA-11 can enhance the radiosensitivity of HPV-positive HNSCC by inducing estrogen receptor α transcription (92). On the other hand, as listed above, the expression of lncUCA1 (101) and lncWISP1 (94) probably exerts a regulatory role in radiation resistance. Sannigrahi et al. (18) have identified that lncWISP1 could activate DNA damage repair and trigger radiation resistance of HPV-negative HNSCC by inhibiting apoptosis-associated protein Bcl-xl and upregulating PI3K kinase. Generally, lncRNAs represent potential targets to overcome chemo- and radiotherapy resistance in HNSCC.

### 3.3 The correlation between E6/E7 and lncRNAs in HPV-positive HNSCC

As previously demonstrated, some lncRNAs have been related to E6 or/and E7 (16, 95). Currently, the regulatory modalities between oncoproteins and lncRNAs have been mainly elucidated in HPV-positive cervical cancer. These mechanisms remain a great enigma and deserve deep exploration in HPV-positive HNSCC. Barr et al. (102) have confirmed that the expression of GAS5, H19, and FAM83H-AS1 was modulated by E6 in cervical cancer. Liu et al. (103) and Zhang et al. (104) have identified that lnc-FANCI-2, HOTAIR, lncPVT1, MALAT1, SNHG12, lnc-CCDST, LINC01101, and LINC00277 were induced by E7, and MALAT1, CCEPR, and TMPOP2 were reciprocally regulated by E6 and E7. Moreover, Jeffers et al. (105) and Ghafouri et al. (16) have reported that MALAT1 and HOTAIR were modulated by E6 and E7 in HPV-positive tumors, respectively.

Sharma et al. (106) have reported the regulatory mechanism of viral oncoproteins on lncRNAs, in which E6/E7 might bind lncRNAs directly or indirectly, thereby impeding their interaction with downstream miRNAs or molecules, involved in biological processes. For example, E7 has been reported to downregulate HOTAIR thereby impeding the repression of HOXD10, which is involved in tumorigenesis and metastasis in SiHa and Caski cells (107). Liu et al. (108) have identified that MALT1 was upregulated by E6, which could act as a molecular sponge for miRNA-124 in the progression of SiHa and Hela cells. Tornesello et al. (109) and Sharma et al. (106) have revealed that the expression of MALAT1 and HOTAIR was related to E7, and the overexpression of MALAT1 contributed to cell proliferation and invasion in HPV-positive HNSCC cells (95). This mechanism has been reported in cervical cancer, showing that E7 upregulated HOTAIR by upregulating miR-214-3p, resulting in the activation of Wnt/β-catenin signaling pathway (110). MALAT1 is upregulated by E7, which promotes the expression of SP1, thereby enhancing the ability of cervical cancer to metastasize (111). The mechanism is possibly similar in HPV-positive HNSCC. However, it has not been proven yet. Further research should be conducted to clarify the regulatory mechanism between viral oncoproteins and lncRNAs.

## 4 The expression and function of circRNA in HPV-positive HNSCC

CircRNA is a kind of circular closed ncRNA lacking 5’-cap and 3’-poly(A) tails, which derived from exons and/or introns of precursor messenger RNA (pre-messenger RNA) (112). The predominant function of circRNAs is acting as molecular sponges, competing with miRNAs or RNAs to regulate biological processes (113). Aberrant expression of circRNAs is responsible for tumor formation, invasion, and metastasis (114, 115). Tornesello et al. (109) have confirmed that the expression of circRNAs was possibly related to oncoproteins expression, which induced the development of HPV-positive tumors. Jun et al. (116), and Chen et al. (117) have confirmed that circRNAs (circ0001971 and circ0001874) were distinctively expressed in OSCC (**Supplemental Table 6**), which had a relatively higher prevalence of HPV infection compared with tumors of other anatomic sites. Zhao et al. (118) have suggested that circRNA expression might have broader relevance to viral oncoproteins. Bonelli et al. (119) have confirmed that circRNAs were involved in tumorigenesis, cancer progression, and chemotherapy resistance, and some of them were related to the TNM stage, which could serve as useful diagnostic and prognostic markers in OSCC.

Aberrant expression of circRNAs is responsible for the clinical behavior of OSCC. Zhao et al. (120) have confirmed that among 32 distinctively expressed circRNAs in OSCC, circ0001874 was correlated with tumor grade, and circ0001971 was correlated with the TNM stage. The researchers have identified that circ0001874 and circ0001971 served as biomarkers of prognosis. Cristóbal et al. (121) have also confirmed that the expression of circUHRF1 and circ0059655 was associated with malignant proliferation, and that of circUHRF1 and circ0001742 was related to migration. However, the expression of circ0001971 (122, 123), circ0005379, and circ0007059 was associated with cisplatin and cetuximab resistance in OSCC (124, 125). These findings indicate that circRNAs could be useful predictors of clinical outcomes in HPV-positive OSCC.

Furthermore, circRNAs may play a regulatory role in tumorigenesis. Zhao et al. (123) have reported that some circRNAs, including circ0002185 and circ0001821, promoted oncogenesis, while other circRNAs, including circ0002203 and circ0004491 suppressed tumorigenesis (126, 127) (**Supplemental Table 6**). CircRNAs play a regulatory role by sponging miRNAs, which concurs with the deregulation of target genes. According to Bonelli et al. (119), circPVT1 repress the expression of miR-497-5p, leading to cell proliferation in HPV-negative OSCC. Furthermore, the high expression of circ0055538 inhibits the cell migration and invasion by regulating the p53/Bcl2/caspase signaling pathway in HPV-negative OSCC (128), indicating that circRNAs could act as targets to intervene in tumorigenesis.

Discrepantly expressed circRNAs have been related to the expression of viral oncoproteins, Zhao et al. (118) and Yu et al. (129) have identified that the expression of circE7 was positively related to E7 in Caski cells, but the regulatory relationship was not verified. Also, a few studies are available on the regulatory relationship of circRNA and viral oncoproteins. The regulatory role research of circRNAs is in a nascent stage in HPV-positive HNSCC, therefore, relevant research is urgently required. It is significant to screen diagnostic and prognostic biomarkers and provide novel insight into biological features of HNSCC from the perspective of circRNA-miRNA-mRNA.

## 5 PiRNAs and HPV-positive HNSCC

PiRNA is a new class of ncRNAs with a length of 26-30nt (130), which generally appears in clusters, but its generating mechanism is still inconclusive (131). PiRNAs can interact with PIWI protein to form piRNA/PIWI protein complexes that silence downstream molecules, which participate in cellular biological activities. Aberrant expression of piRNAs is responsible for the occurrence of malignancy. According to Firmino et al. (132), HPV status might affect the expression of piRNAs, among which 30 piRNAs and 11 piRNAs were confirmed to be distinctively expressed in HPV-positive (n=83) and HPV−negative HNSCC tissues (n=370), respectively. Krishnan et al. (133) have also identified a total of 30 differently expressed piRNAs in HPV-positive HNSCC samples compared with their counterparts.

The significance of discrepantly expressed piRNAs is that they can act as prognostic biomarkers (133, 134). Krishnan et al. (133) have found that the level of NONHSAT077364, NONHSAT144936, and NONHSAT054230 displayed a close relationship with pathologic stage and nodal metastasis in HPV-positive HNSCC. Discrepant expression of piRNAs play a regulatory role in oncogenesis in HPV-positive HNSCC. Researchers have observed that NONHSAT059231 and NONHSAT077463 are correlated with oncogenesis in HNSCC (132) (**Supplemental Table 7**). Additionally, NONHSAT069719 inhibited the tumorigenesis of HPV-positive HNSCC, other piRNAs, including NONHSAT077364, NONHSAT102574, and NONHSAT128479 promoted tumor pathogenesis and progression in HPV-positive HNSCC (133). Further research has shown that piRNAs executed their functionality by associating with PIWI proteins (PIWIL1-PIWIL4), which could enhance cell proliferation (133, 135). As mentioned above, some piRNAs are distinctively expressed in HPV-positive HNSCC, which is probably induced by HPV infection. However, rigorous studies on the regulatory relationship between piRNA and viral oncoproteins are lacking, thus, requiring additional exploration.

## 6 SnoRNAs and HPV-positive HNSCC

SnoRNA is a category of ncRNA with a length of 60-300nt that mainly exists in nucleosomes, and is generally used for the synthesis and modification of ribosomal RNA and mRNA (136). SnoRNAs are also involved in the proliferation and apoptosis of tumor cells (137). Xing et al. (138) have identified that the expression of snoRNAs was associated with clinical features of HNSCC, and distinctive expression of snoRNAs in HNSCC was associated with histological grade and tumor progression. For example, SNORD114‐17 was involved in the regulation of cell adhesion, invasion, and metastasis, and U3 (chr2) was related to RNA editing (138). Furthermore, according to Xing et al. (138), the expression of SNORD114-17 (ENSG00000201569), SNORA36B (ENSG00000222370), SNORD78 (ENSG00000212378), ENSG00000212182, and ENSG00000212195 was related to clinical stages, histological grade, T classification, lymph node metastasis and anatomic subdivisions, indicating that snoRNAs could serve as biomarkers of HNSCC. However, there are only several related reports of snoRNAs in HNSCC. Hence, the relationship between snoRNAs and HPV infection remains unexplored. Moreover, their expression and regulatory role have not been reported in HPV-positive HNSCC. It is possible that the expression and regulatory mechanisms of snoRNAs could pave the way to tumorigenesis and clinical characteristics of HPV-positive HNSCC.

## 7 Discussion

NcRNAs include miRNAs, lncRNAs, circRNAs, and piRNAs (139). Deregulated ncRNAs in HNSCC have been related to HPV infection, which probably exerts regulatory role in the clinical-pathological features of the tumor (**Supplemental Table 8**).

Besides, the discrepant expression of ncRNAs can be distinguished in HPV-positive and HPV-negative HNSCC. Specifically, miRNAs and lncRNAs are different in sample sources (tumor tissues/cell lines), regulation oncoproteins (E6/E7), regulation status (upregulated/downregulated), and effects (biological process/clinical characteristics) in HPV-positive HNSCC. Furthermore, these miRNAs/lncRNAs are involved in different modulation mechanisms, some of them can serve as early molecular markers for the diagnosis and prognosis (17). Also, ncRNAs may become effective targets for tumor suppressor drugs. Currently, the main treatment for HNSCC is surgery combined with chemoradiotherapy, suggesting that ncRNAs could be the targets for improving the sensitivity of HPV-positive HNSCC to chemoradiotherapy. NcRNAs with oncogenic effects may become effective targets for tumor intervention drugs. However, up to now, only a part of the biological functions and regulatory roles of ncRNAs has been clarified. NcRNAs can regulate the characteristics of HPV-positive HNSCC through joint action (140, 141). The interaction network of ncRNAs should be constructed to further reveal the interaction among different types of ncRNAs, thus, providing diagnostic and therapeutic targets for HPV-positive HNSCC. In summary, we mainly focusd on the expression profile of ncRNAs (including lncRNA, miRNA, and circRNA) and explored their regulatory role and interconnection in HPV-positive HNSCC, aiming to clarify the regulatory mechanism of ncRNAs in HPV-positive HNSCC. However, the research on the modulation mechanism of E6/E7 on miRNAs, lncRNAs, and circRNAs is still in its infancy in HPV-positive HNSCC. Further investigation is required to elucidate the biological functions and regulatory roles of ncRNA in HPV-positive HNSCC. Nevertheless, ncRNAs seem to possess therapeutic prospects, therefore, more preclinical studies *in vitro* and *in vivo* are necessary to explore effective targeted therapies with a view to improving the prognosis of HPV-positive HNSCC.

## Author contributions

DG and CZ: conception and design. All authors contributed to the article and approved the submitted version.
