# Supplementary material for: Expression and molecular regulation of non-coding RNAs in HPV-positive head and neck squamous cell carcinoma
Source: Front Oncol. 2023 Mar 29;13:1122982. doi: 10.3389/fonc.2023.1122982 (PMC10090466; doi:10.3389/fonc.2023.1122982)
Supplement: Supplementary file 1 [file Table_1.docx]

|  | **Table1. Distinctively expressed miRNAs in HPV-positive HNSCC** | | | | | | | | | |
| --- | --- | --- | --- | --- | --- | --- | --- | --- | --- | --- |
| **Authors** | **miRNA ID** | | | **Samples origin** | | **Samples types** | **Detection methods** | | **Expression status** | |
| Božinović et al. (28) | miR-100-5p, miR-218-5p  miR-27a-5p, miR-31-5p,  miR-9-5p, miR-21-3p, miR-34a-5p | | | HPV-positive oropharyngeal cancer | | Fresh-frozen tissues | Next generation sequencing | | Overexpression | |
|  | miR-143-3p, miR-145-5p | | | HPV-positive oropharyngeal cancer | | Fresh-frozen tissues | Next generation sequencing | | Decreased  expression | |
| Nunvar et al.  (35) | miR-101-3P, miR-10b-5p,  miR-29c-3p, miR-30a-5p, miR-451a, miR-195-5p,  miR-663a, miR-142-5p,  miR-574-3p | | | HPV-positive TSCC | | FFPE samples | Whole-genome  bisulfite sequencing | | Decreased  expression | |
|  | miR-4800-3p, miR-3196,  miR-210-3P, miR-769-5p,  miR-1307-5p | | | HPV-positive TSCC | | FFPE samples | Whole-genome  bisulfite sequencing | | Overexpression | |
| Vojtechova et al. (40) | miR-944, miR-1825,  miR-135, miR-210,  miR-1180, miR-205,  miR-643, miR-335,  miR-1244, miR-17,  miR-27, miR-183  miR-224, miR-8 | | | HPV-positive TSCC | | Fresh frozen tonsillar cancer tissues | RT-PCR | | Overexpression | |
| Vojtechova et al. (40) | miR-221, miR-26,  miR-30, miR-577,  miR-29, miR-15,  miR-101, miR-143,  miR-486, miR-199,  miR-140, miR-204,  miR-145, miR-139,  miR-144, miR-184 | | | HPV-positive TSCC | | Fresh frozen tonsillar cancer tissues | RT-PCR | | Decreased  expression | |
| Wan et al. (41) | | miR-134, miR-196a, miR-210, miR-455 | HPV-positive HNSCC | | Saliva samples | | | RT-PCR | | Overexpression |
| Lajer et al. (42) | miR-150, miR-146b-5p,  miR-21, miR-363,  miR-15a, miR-34a,  miR-20b, miR-146a, miR-let-7g, miR-let-7f, miR-625, miR-155,  miR-15b, miR-29a,  miR-125b, miR-26b,  miR-342-3p, miR-768-3p | | | HPV-positive HNSCC | | Fresh frozen samples | Microarray analysis | | Overexpression | |
|  | miR-99b, miR-877,  miR-744, miR-1180,  miR-31, miR-193b | | | HPV-positive HNSCC | | Fresh frozen samples | Microarray analysis | | Decreased  expression | |
| Gougousis et al. (45) | miR-15a, miR-16,  miR-143, miR-145,  miR-106-363 | | | HPV-positive OPSCC | | Fresh-frozen tissues | Whole-genome bisulfite sequencing | | Unknown | |
|  |  |  |  |  |  |  |  |  |  |  |
| Lajer et al. (46) | miR-127-3p, miR-379, miR-125a-5p, miR-432,  miR-409-5p, miR-433,  miR-381, miR-199a-3p, miR-26b, miR-199b-5p,  miR-1201, miR-126,  miR-409-3p, miR-101,  miR-143, miR-145,  miR-517a | | | HPV-positive PSCC | | Fresh-frozen tissues | MiRNA microarray analysis  RT-PCR | | Decreased  expression | |
|  | miR-195, miR-363 | | | HPV-positive PSCC | | Fresh-frozen tissues | MiRNA microarray analysis  RT-PCR | | Overexpression | |
| House et al. (49) | miR-127-3p, miR-363 | | | HPV-positive OPSCC | | Fresh-frozen tissues  Cell lines | RT-PCR | | Overexpression | |
|  |  |  |  |  |  |  |  |  |  |  |
| Wald et al. (54) | miR-363, miR-33,  miR-497 | | | HPV-positive HNSCC | | Cell lines | RT-PCR | | Overexpression | |
|  | miR-155, miR-181a,  miR-181b, miR-29a,  miR-218, miR-222,  miR-221, miR-142-5p | | | HPV-positive HNSCC | | Cell lines | RT-PCR | | Decreased  expression | |
| Miller et al. (56) | miR-199a-3p, miR-143,  miR-145, miR-126a, miR-125a, miR-31,  miR-199b, miR-126,  miR-193b | | | HPV-positive OPSCC | | Fresh-frozen tissues | RT-PCR | | Decreased  expression | |
|  | miR-9, miR-15b,  miR-162, miR-20b,  miR-25, miR-29c, miR-93, miR-106a, miR-106b, miR-107, miR-148a, miR-150, miR-222, miR-320a, miR-335, miR-363,  miR-378, miR-598,  miR-625 | | | HPV-positive OPSCC | | FFPE samples  cell lines | RT-PCR  sequencing | | Overexpression | |

Footnote: HNSCC: Head and neck squamous cell carcinoma, TSCC: Tongue squamous cell carcinoma, OSCC: Oral squamous cell carcinoma, PSCC: Pharyngeal squamous cell carcinoma, OPSCC: Oropharyngeal squamous cell carcinoma, FFTE: Formalin-Fixed and paraffin-Embedded, RT-PCR: Real-time quantitative PCR
