# Supplementary material for: Expression and molecular regulation of non-coding RNAs in HPV-positive head and neck squamous cell carcinoma
Source: Front Oncol. 2023 Mar 29;13:1122982. doi: 10.3389/fonc.2023.1122982 (PMC10090466; doi:10.3389/fonc.2023.1122982)
Supplement: Supplementary file 2 [file Table_2.docx]

| **Table 2. MiRNAs-related clinical features in HPV-positive HNSCC** | | | |
| --- | --- | --- | --- |
| **Authors** | **miRNA ID** | **Sample origin** | **Clinical features** |
| Tumban et al. (21) | miRNA-133a | HNSCC tissues (HPV-positive) | tumorigenesis suppression |
| Salazar et al. (22) | miR-92a-3p, miR-124-3p, miR-122-5p, miR-146a-5p | OPSCC tissues (HPV-positive) | TNM stages |
|  | miR-122-5p | OPSCC tissues (HPV-positive) | prognosis (positively) |
| Emmett et al.& Gougousis et al (23, 45) | miR-21 | HNSCC tissues (HPV- positive) | radiosensitivity |
| Wan et al. (41) | miRNA-9,  miR-127,  miR-196a,  miR-196b,  miR-222,  miR-455 | HNSCC patients (HPV-positive) | TNM stages |
| Lajer et al. (46) | miR-125b | HNSCC tissues (HPV-positive) | proliferation, cell motility,  and invasiveness suppression |
| House et al. (49) | miR-133a-3p | OPSCC tissues (HPV-positive) | lymph node metastasis |
| Weiss et al. (50) | miR-205-5p, miR-182-5p | OPSCC tissues (HPV-positive) | prognosis (negatively) |
| Bersani et al. (51) | miR-363,  miR-127-3p,  miR-155 | HNSCC tissues (HPV-positive) | prognosis (positively) |
|  | miR-185 | HNSCC tissues (HPV- positive) | prognosis (negatively) |
|  | miR-193b | HNSCC tissues (HPV-positive) | high T-stage. |
| Zhang et al. (53) | miR-106a | HNSCC cells (HPV-positive) | radiosensitiviy |
| Miller et al. (56) | miR-93,  miR-20b,  miR-363,  miR-106b, | OPSCC tissues (HPV-positive) | proliferation and invasion inhibition |
| Hess et al. (60) | miR-155 | HNSCC tissues (HPV-positive) | low T-stage |
| Long et al. (69) | miR-27 | HNSCC tissues (HPV-positive) | prognosis (positively) |
| Inoue et al. (71) | miR-137,  miR-296,  miR-149,  miR-365-2,  miR-130b | HNSCC tissues (HPV-positive) | radioresistance |
| Lu et.al. (74) | miR-15a | HNSCC tissues (HPV-positive) | prognosis (positively) |

Footnote: OPSCC: Oropharyngeal squamous cell carcinoma, HNCC: Head and neck squamous cell carcinoma.
