# Supplementary material for: Expression and molecular regulation of non-coding RNAs in HPV-positive head and neck squamous cell carcinoma
Source: Front Oncol. 2023 Mar 29;13:1122982. doi: 10.3389/fonc.2023.1122982 (PMC10090466; doi:10.3389/fonc.2023.1122982)
Supplement: Supplementary file 3 [file Table_3.docx]

| **Table 3. MiRNAs modulated by viral oncoproteins in HPV-positive tumors** | | | | | |
| --- | --- | --- | --- | --- | --- |
| **Authors** | **miRNA ID** | **Samples Origin** | **Viral oncoprotein** | **Status** | **Effects** |
| Wang et al. (13) | miR-19 | CC cells (HPV-positive) | E6 | downregulated | unknown effects |
| Sannigrahi et al. (18) | miR-17-92a, miR-378 | CC cells (HPV-positive) | E7 | upregulated | promoting malignant proliferation |
|  | miR-20,  miR-18 | CC cells (HPV-positive) | E7 | downregulated | unknown effects |
|  | miR-139-3p | HNSCC cells  (HPV-positive) | E6/E7 | unknown | promoting chemotherapy sensitivity (cisplatin and 5-fluorouracil) |
|  | miR-23a,  miR-26a | HNSCC cells  (HPV-positive) | E6 | upregulated | unknown effects |
|  | miR-17-5p | CC cells (HPV-positive) | E6 | downregulated | unknown effects |
|  | miR-15b | CC cells (HPV-positive) | E7 | upregulated | promoting malignant proliferation |
|  | miR-16 | CC cells (HPV-positive) | E7/E6 | upregulated | promoting tumorigenesis |
| Salazar et al. (22) | miR-92a | OPSCC cells  (HPV-positive) | E7/E6 | upregulated | promoting malignant proliferation |
|  | miR-25 | OPSCC cells  (HPV-positive) | E7 | upregulated | promoting malignant proliferation |
| Gunasekharan et al. (32) | miR-145 | CC cells with  HPV infection | E7 | downregulated | unknown effects |
| Emmett et al. (33) | miR-29a | HNSCC cells  (HPV-positive) | E7/E6 | downregulated | suppressing tumorigenesis |
|  | miR-21 | HNSCC cells  (HPV-positive) | E7 | upregulated | promoting chemotherapy resistance |
| Lajer et al. (42) | miR-15a | HNSCC cells  (HPV-positive) | E7 | upregulated | promoting malignant proliferation |
|  | miR-16 | HNSCC cells  (HPV-positive) | E7 | downregulated | suppressing malignant proliferation |
|  | miR-17 | HNSCC cells  (HPV-positive) | E7 | downregulated | suppressing malignant proliferation |
|  | miR-363 | TSCC cells  (HPV-positive) | E6 | upregulated | attenuating migration |
|  | miR-181a | HNSCC cells  (HPV-positive) | E6 | downregulated | unknown effects |
|  | miR-29a. | HNSCC cells  (HPV-positive) | E6 | downregulated | unknown effects |
|  | miR-218 | HNSCC cells  (HPV-positive) | E6 | downregulated | unknown effects |
|  | miR-100 | CC cells (HPV-positive) | E7/E6 | downregulated | promoting tumorigenesis |
|  | miR-22  miR-27a | CC cells (HPV-positive) | E7 | downregulated | promoting tumorigenesis |
| Lajer et al.  & Wang et al.  (13, 42) | miR-34a | CC cells (HPV-positive) | E7/E6 | downregulated | suppressing tumorigenesis |
| Zhang et al. (53) | miR-106a | HNSCC cells  (HPV-positive) | E7 | upregulated | promoting radiotherapy sensitivity |
| Long et al. (69) | miR-27a | HNSCC cells  (HPV-positive) | E6 | upregulated | promoting radiotherapy sensitivity |

Footnote: CC: Cervical carcinoma, HNSCC: Head and neck squamous cell carcinoma, OPSCC: Oropharyngeal Squamous Cell Carcinoma, TSCC:Tongue squamous cell carcinoma.
