# Supplementary material for: Expression and molecular regulation of non-coding RNAs in HPV-positive head and neck squamous cell carcinoma
Source: Front Oncol. 2023 Mar 29;13:1122982. doi: 10.3389/fonc.2023.1122982 (PMC10090466; doi:10.3389/fonc.2023.1122982)
Supplement: Supplementary file 4 [file Table_4.docx]

**Table 4. Distinctively expressed lncRNAs in HPV-positive HNSCC**

| **Authors** | **LncRNA ID** | **Samples origin** | **Samples type** | **Detection methods** |
| --- | --- | --- | --- | --- |
| Ma et al. (82) | nc_NONHSAG010914, nc_NONHSAT018263, nc_NONHSAT083749,  nc_NONHSAT040523, nc_NONHSAG048989, nc_NONHSAT040500,  nc_NONHSAT095654,  nc_NONHSAT122146,  nc_NONHSAT006502  NONHSAG-011264,  NONHSAT008740,  LINC01305, FR302050, nc_NR_029467.1 | HNSCC tissues  (HPV-positive) | Fresh-frozen tumor samples | RT-PCR,  lncRNA microarray |
| Nohata et al. (85) | LINC01089, PTOV1-AS1, LOC285889, UNC58-AS1, FAM182A, PTOV1-AS1,  LOC285889, TPRG1-AS2, LINC00551,  LINC01011, LINC01122, LINC00582,  LINC00314, LOC644848, DNM1P35,  MTUS2-AS1, LINC00928, TTTY10, TTTY21, | HNSCC cells  (HPV-positive) | TCGA samples HNSCC cell lines | RNA sequencing |
| Yang et al. (87) | LNCRNA U62317.3,  LNCRNA KLHDC7B,  LINC00662 | HNSCC samples  (HPV-positive) | TCGA tumor samples | Bioinformatic analysis tools (package of R software) |
| Haque et al. (88) | HEIH, LUCAT1, LUCAT1,  LINC00152, HAND2-AS1,  MEG3, TERC | HNSCC samples  (HPV-positive) | TCGA samples | TCGA-RNA-seq |
| Kolenda et al. (90) | EGOT | HNSCC samples  (HPV-positive) | TCGA samples | Bioinformatic tools  (DAVID 6.7) |

Footnote: RT-PCR: Real-time quantitative PCR, HNSCC: Head and neck squamous cell carcinoma, TCGA: The Cancer Genome Atlas.
