# Supplementary material for: Expression and molecular regulation of non-coding RNAs in HPV-positive head and neck squamous cell carcinoma
Source: Front Oncol. 2023 Mar 29;13:1122982. doi: 10.3389/fonc.2023.1122982 (PMC10090466; doi:10.3389/fonc.2023.1122982)
Supplement: Supplementary file 5 [file Table_5.docx]

| **Table 5. The distinctive expression and role of lncRNAs in HPV-HNSCC** | | | | | | | |
| --- | --- | --- | --- | --- | --- | --- | --- |
| **Authors** | **LncRNAs ID** | | **Samples origin** | | **Function/Effect** | |  |
| Sannigrahi et al. (18) | LNC01089, LNC01305,  PTOV1-AS1 | | HNSCC tissues  (HPV-positive) | | regulating tumorigenesis | |  |
|  | HOTAIR  PROM1  CCAT1  MUC19 | | HNSCC tissues  (HPV-positive) | | promoting myeloid-derived suppressor cell recruitment of HNSCC | |  |
| Dai et al. (80) | LNCRNA BLACAT1 | | OSCC tissues  (HPV-positive) | | promoting malignant proliferation and invasion | |  |
| Ma et al.(82) | nc_NONHSAG010914, nc_NONHSAT083749, nc_NONHSAT018263, nc_NONHSAT040523, nc_NONHSAG048989, nc_NONHSAT040500, nc_NONHSAT095654, nc_NONHSAT122146, nc_NONHSAT006502,  nc_NR_029467.1 | | HNSCC tissues  (HPV-positive) | | regulating tumorigenesis | |  |
| Kopczyńska et al. (83) | H19 | | HNSCC tissues  (HPV-positive) | | tumorigenesis and epithelial-mesenchymal transition. | |  |
|  | PRINS, TTTY14,  TTTY15, CDKN2B-AS1 | | HNSCC tissues  (HPV-positive) | | prognosis (positively) | |  |
|  |  |  |  |  |  |  |  |
|  |  |  |  |  |  |  |  |
|  |  |  |  |  |  |  |  |
| Wang et al. (86) | LINC00520, LINC00460, LINC00487 | | HNSCC tissues  (HPV-negative) | | prognosis (negatively) | |  |
|  |  |  |  |  |  |  |  |
|  |  |  |  |  |  |  |  |
| Fang et al. (93) | MEG3 | | HNSCC tissues  (HPV-negative) | | tumor size, lymph node metastasis, prognosis, and invasiveness | |  |
|  | LNCUCA1 | | HNSCC tissues  (HPV-negative) | | chemotherapy resistance | |  |
| Kolenda et al. (90) | EGOT | | TCGA HNSCC samples (HPV-positive) | | prognosis (positively) | |  |
| Haque et al. (88) | | HAND2-AS1  SPRY4-IT1 | | HNSCC tissues  (HPV-negative) | | prognosis (negatively) |  |
| Song et al. (92) | LNC02535,  LNC-SERPINB12-3,  LNC-LGALS7B-1,  CCDC144NL-AS1 CD81-AS1, LNC-KLF7-1,  LNC00504, RP11-635N19.3, AC007879.2, RP11-30P6.6,  AC006946.16, AC104-534.2,  LNC-IL17RA-11 | | HNSCC tissues  (HPV-positive) | | prognosis (positively) | |  |
| Zhang et al.(94) | LNCWISP1 | | HNSCC tissues  (HPV-negative) | | radioresistance | |  |
| Dias et al. (95) | H19 | | HNSCC tissues  (HPV-positive) | | inhibiting invasion  and metastasis | |  |

Footnote: HNSCC: Head and neck squamous cell carcinoma, OSCC: Oral squamous cell carcinoma, TCGA: The Cancer Genome Atlas.
