# Supplementary material for: Expression and molecular regulation of non-coding RNAs in HPV-positive head and neck squamous cell carcinoma
Source: Front Oncol. 2023 Mar 29;13:1122982. doi: 10.3389/fonc.2023.1122982 (PMC10090466; doi:10.3389/fonc.2023.1122982)
Supplement: Supplementary file 6 [file Table_6.docx]

| **Table 6. CircRNAs related clinical features and regulatory role in OSCC/TSCC** | | | | |
| --- | --- | --- | --- | --- |
| **Authors** | **CircRNA ID** | **Samples types** | **Samples origin** | **Functions/Effects** |
| Zhao et al. & Chen et al. (117, 118) | Circ_0086414, Circ_0002185, Circ_0092125, CircMAN1A2, Circ_0072387, Circ_0001821,  Circ_0008309, Circ_001242, Circ_0001874, Circ_0001971 | tissues | OSCC | diagnosis |
|  | CircUHRF1, Circ_0001742,  Circ_0059655, Circ_0001971, Circ_0002203, Circ_100290, Circ_0070401, CircFLNA, Circ_0007059, Circ_0063772,  CircPVT1, CircHIPK3 | tissues | OSCC | promoting proliferation |
| Cristóbal et al.(121) | CircDOCK1 | tissues and cells | OSCC | promoting tumorigenesis |
| Cristóbal et al.(121) | Circ_0002185, Circ_0001821, Circ_100290, Circ_0001742, Circ_0059655, Circ_0002203,  Circ_000449, Circ_0063772, Circ_0070401, Circ_0005379, CircFLNA, Circ_0007059, Circ_0012342 | tissues and cells | OSCC | promoting oncogenesis |
| Hu et al.& Zhao et a l. & Su et al. & Li et al (122-128) | CircUHRF1, Circ_0001742,  Circ_0001971, Circ_0002203, Circ_0004491, Circ_0059655, CircFLNA, Circ_0063772, Circ_0005379, Circ_0007059  Circ_0070401 | tissues | OSCC  TSCC | migration and invasion |
|  | CircUHRF1, Circ_0001742,  Circ_0004491, Circ_0005379 | cells | OSCC | EMT |
|  | Circ_0001971, Circ_0005379 | cells | OSCC | drug resistance (cisplatin, cetuximab) |
|  | CircDOCK1 | tissues and cells | OSCC | promoting tumorigenesis |

| Hu et al.& Zhao et a l. & Su et al. & Li et al (122-128) | Circ_0002185, Circ_0001821, Circ_100290, Circ_0001742, Circ_0059655, Circ_0002203,  Circ_0004491, Circ_0063772, Circ_0070401, Circ_0005379, CircFLNA, Circ_0007059, Circ_0012342 | tissues and cells | OSCC | promoting oncogenesis |
| --- | --- | --- | --- | --- |
| Zhao et al. (123) | circUHRF1 | cells | OSCC | promoting proliferation, migration, invasion, and EMT |
| Su et al. (125) | circ_0007059 | cells | OSCC | suppressing cell growth, migration, and invasion, facilitating apoptosis |

Foot note: OSCC: Oral squamous cell carcinoma; TSCC: Tongue squamous cell carcinoma; EMT: Epithelial-to-mesenchymal transition.
