# Supplementary material for: Expression and molecular regulation of non-coding RNAs in HPV-positive head and neck squamous cell carcinoma
Source: Front Oncol. 2023 Mar 29;13:1122982. doi: 10.3389/fonc.2023.1122982 (PMC10090466; doi:10.3389/fonc.2023.1122982)
Supplement: Supplementary file 7 [file Table_7.docx]

| **Table 7. The expression and mole of piRNAs in HPV-positive HNSCC** | | | |
| --- | --- | --- | --- |
| **Authors** | **PiRNAs ID** | **Samples origin** | **Functions/Effects** |
| Casarotto et al. (15) | FR018916, FR197104,  FR29875, FR140858, FR2371807 | HNSCC tissues  (HPV-positive) | prognosis |
|  | PiR-30652 | HNSCC tissues  (HPV-positive) | higher histologic grade |
| Firmino et al. (132) | NONHSAT077364  NONHSAT144936 | HNSCC tissues  (HPV-positive) | tumor size |
|  | NONHSAT144936  NONHSAT077364  NONHSAT059231 | HNSCC tissues  (HPV-positive) | promoting malignant proliferation. |
|  | FR018916, FR140858,  FR197104, FR237180, FR298757 | HNSCC tissues  (HPV-positive) | prognosis (positively) |
|  | NONHSAT054230 | HNSCC tissues  (HPV-positive) | lymph node metastasis |
| Krishnan et al. (133) | NONHSAT102574, NONHSAT128479 | HNSCC tissues  (HPV-positive) | promoting the proliferation and invasion. |
|  | NONHSAT069719, NONHSAT108298 |  |  |
| Zou et al. (134) | PiR-34736 | HNSCC tissues  (HPV-positive) | prognosis (positively) |

Footnote: HNSCC: Head and neck squamous cell carcinoma.
