# Supplementary material for: Expression and molecular regulation of non-coding RNAs in HPV-positive head and neck squamous cell carcinoma
Source: Front Oncol. 2023 Mar 29;13:1122982. doi: 10.3389/fonc.2023.1122982 (PMC10090466; doi:10.3389/fonc.2023.1122982)
Supplement: Supplementary file 8 [file Table_8.docx]

**Table8 The modulation role of target genes in non-coding RNAs in HPV-positive HNSCC**

| **Authors** | **MiRNA** | **Target genes** | **Target role** |
| --- | --- | --- | --- |
| Sannigrahi et al. (18) | miR-211 | unknown | Carcinogenesis promotion |
|  | miR-155 |  |  |
| Salazar et al. (22) | miR-92a | unknown | Malignant proliferation promotion |
| Emmett et al. (23) | miR-21 | unknown | Chemoradiotherapy resistance |
| Wang et al. (37) | miR-107 | *AV1, CDK6, MYB, and SERPINB5* | Modulating P53 signaling pathway, PI3K-Akt signaling pathway, and autophagy pathway |
|  | miR-142-3p | *PPFIA1* | Implicated in HPV-induced tumorigenesis, and transcriptional dysregulation |
|  | miR-9 | *NOX4* | Promoting fibroblast phenotype |
| Lajer et al. (42) | miR-363 | *BCL2L11, CDKN1A, HI VEP1, CASP3, CD274* (predicted by miRtarbase) | Apoptosis, cell cycle, and immunology modulation |
|  | miR-26b | *PTGS2, EPHA2, CCNE1, TAB1, RB1*  (predicted by miRtarbase) | Prostaglandin biosynthesis,  cell cycle, TGF-beta-interleukin 1  and WNT-1-signaling modulation |
|  | miR-29a | *MCL1, DNMT3A, DNMT3B, BCL2, PIK3R1* | Apoptosis, DNA methylation,  and PI3K-signaling modulation |
|  | miR-15a | unknown | Malignant proliferation promotion |
|  | miR-125a | *ERBB3, CDKN1A, CD34, TP53, ERBB2* | Cell cycle modulation, EGF signaling,  cell attachment, and tumor suppression |
| Lajer et al.& Miller et al.  (42, 56) | miR-199a | *MET, MTOR, GSK3B, WNT2, HIF1A* | DNA damage, nutrient deprivation,  cell cycle arrest, immunosuppressive effects, transformation, energy metabolism,  body pattern formation, oncogenesis,  cell fate, embryogenesis |
|  | miR-126 | *VEGFA, SOX2, KRAS, PIK3R2, TERT* | Proliferation and migration of vascular endothelial cells, cell fate, stem cell maintenance, transformation, EMT, PI3K-signaling, telomere elongation |
| Lajer et al. & Gao et al. (42, 58) | miR-31 | *RHOA, SATB2, FOXP3, MMP16, HIF1AN* | Tumor cell proliferation and Metastasis, transcription regulation,  chromatin remodeling,  and immunology modulation |
| Zhang et al. (53) | miR-106a | *RUNX3* | Promoting radiation sensitivity |
| Wald et al. (54) | miR-155 | *CEBPB, TAB2, TP53INP1, SMAD1, KRAS* | Immune and inflammatory response,  TGF-beta-TP53-signaling modulation,  cell growth, apoptosis, morphogenesis, development, and immune response |
|  | miR-222 | *CDKN1B, MMP1, KIT, PTEN, CDKN1C* | Cell cycle modulation and tumor suppression |
| Miller et al. (56) | miR-143 | *KRAS, MAPK7, MYO6, DNMT3A, FNDC3B* | Proliferation, differentiation, transcription regulation, intracellular vesicle and organelle transport, and DNA methylation modulation |
|  | miR-145 | *BNIP3, STAT1, FSCN1, KLF5, SOX2* | Apoptosis, cell viability, proliferation, migration, motility, adhesion and cellular interactions, embryonic development, stem cell maintenance, and EMT |
| Liang et al. (65) | miR-27 | *CCL22* | Infiltration of regulatory T cells (Tregs) facilitation |
| Long et al. (69) | miR-27a | *SMG1* | Promoting radiation sensitivity |
| Shiiba et al. (70) | miR-125b | *ICAM2* | Attenuating sensitivity |
| Inoue et al. (71) | miR-130b | *p63* | Promoting radioresistance |

| **Authors** | **LncRNA**  **/CircRNA** | **Target genes/signaling pathway** | **Target function** |
| --- | --- | --- | --- |
| Ma et al. (82) | PROM1 | unknown | Inflammation, immunity, and cancer development modulation |
|  | HOTAIR |  |  |
|  | CCAT1 |  |  |
|  | MUC19 |  |  |
| Kopczyńska et al. (83) | LNC-PRINS | unknown | Inflammatory response, chemokines,  and immune response |
| Yang et al. (87) | KCNQ1OT1 | unknown | Induced cisplatin resistance. |
| Kolenda et al. (90) | EGOT | unknown | Cellular processes (differentiation, adhesion, developmental process, cell communication modulation, and signal transduction) |
| Song et al. (92) | LNCIL17RA-11 | *ER-alpha* | Promoting radiosensitivity |
| Fang et al. (100) | MEG3 | unknown | Invasiveness promotion |
| Barr et al. (102) | LNCFAM83H-AS1(onco-lncRNA-3) | E6-p300 pathway | Carcinogenesis, promotion of cellular proliferation, migration and increased apoptosis |
| Bonelli et al. (119) | Circ_0001821  (CircPVT1) | miR-497-5p (*AURKA*), *BUB1* mitotic checkpoint serine/threonine kinase | Proliferation and colonies formation |
| Hu et al. (122) | Circ_0001742 | miR-634/*RAB1A* | Proliferation, migration, invasion, and EMT |
| Su et al. (128) | Circ_0055538 | *TP53/BCL2*/*CASP3* | Migration and invasion of cells |

Footnote: EMT: Epithelial-to-mesenchymal transition.
